# Solid–solid phase transitions via melting in metals

**DOI:** 10.1038/ncomms11113

**Published:** 2016-04-22

**Authors:** S. Pogatscher, D. Leutenegger, J. E. K. Schawe, P. J. Uggowitzer, J. F. Löffler

**Affiliations:** 1 Laboratory of Metal Physics and Technology, Department of Materials, ETH Zurich, Zurich 8093, Switzerland; 2 Institute of Nonferrous Metallurgy, Department of Metallurgy, Montanuniversität Leoben, Leoben 8700, Austria; 3 Mettler-Toledo GmbH, Analytical, Schwerzenbach 8603, Switzerland

## Abstract

Observing solid–solid phase transitions *in-situ* with sufficient temporal and spatial resolution is a great challenge, and is often only possible via computer simulations or in model systems. Recently, a study of polymeric colloidal particles, where the particles mimic atoms, revealed an intermediate liquid state in the transition from one solid to another. While not yet observed there, this finding suggests that such phenomena may also occur in metals and alloys. Here we present experimental evidence for a solid–solid transition via the formation of a metastable liquid in a ‘real' atomic system. We observe this transition in a bulk glass-forming metallic system *in-situ* using fast differential scanning calorimetry. We investigate the corresponding transformation kinetics and discuss the underlying thermodynamics. The mechanism is likely to be a feature of many metallic glasses and metals in general, and may provide further insight into phase transition theory.

## Abstract (web summary)

Solid–solid phase transition via an intermediate liquid state has been identified in colloidal systems, but the universality of the phenomenon at atomic scales has not yet been proved. Pogatscher *et al.* observe a similar transition in a metallic glass system using fast differential scanning calorimetry.

## Graphical abstract

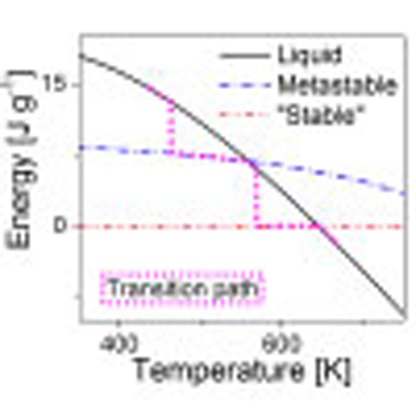

Nearly all classes of materials show solid-state phase transitions. Understanding transformations from one crystal to another is a key topic in materials science because in many cases they determine material properties. The transition from graphite to diamond^1^, the property design of steels^2^ or the formation of metastable phases to strengthen aluminium^3^ are just a few well-known examples of solid–solid transitions in crystalline materials. Solid-state transitions from amorphous to crystalline or even vice versa^4^,^5^ have also been reported in metals, and they are also of great importance in other fields. For example, pharmaceutical substances can drastically alter their bioavailability in the human body via transitions from a metastable to a stable polymorphic modification^6^. These mechanisms are complex and in most cases difficult to investigate via *in-situ* experiments. The atomistic mechanisms of solid–solid phase transitions are therefore still poorly understood. In the past this limitation was circumvented by deploying colloidal model systems^7^, where the motion of particles can be directly observed via video microscopy. Recently, one such study on micron-sized polymer particles discovered an intermediate liquid state in the transition from one solid to another^8^. This suggests that such phenomena may also occur in ‘real' atomic systems such as metals and alloys. However, because of the spatial localization and rapid kinetics of such transitions^9^, this phenomenon has not yet been demonstrated in metallic systems.

Orava *et al.*^10^ illustrated the power of novel chip-based fast differential scanning calorimetry (FDSC) in studying the rapid crystallization of an amorphous Ge_2_Sb_2_Te_5_ phase-change material. In their study, small-scale samples on a chip sensor with low thermal mass facilitated precise measurements of thermodynamic data at high speed^11^,^12^. The slowest known transformation kinetics within metallic materials has been obtained for metallic glasses^13^. The first metallic glass discovered was a Au–Si system^14^. Since then Au-based bulk metallic glasses (BMGs) with very slow crystallization kinetics have been found^15^,^16^, and their suitability for FDSC investigations has recently been demonstrated^17^,^18^. They are also of interest for future applications, for example, in small-scale devices for information technology^19^,^20^, and are known to form various metastable crystalline states^21^.

By deploying a Au_70_Cu_5.5_Ag_7.5_Si_17_ BMG and FDSC we are in the unique situation of being able to measure a slowly transforming metallic system using a very fast detection technique. With this method we have been able to demonstrate unambiguously that a metallic system can transform from a metastable crystalline state into a more stable crystalline state via the formation of a metastable liquid at a temperature far below the deepest equilibrium eutectic temperature. This transformation differs from the usual crystallization pathway of metallic glasses, which often occurs via primary crystallization at low temperature and eutectic crystallization of the residual supercooled liquid (which now has a composition different from that of the original glass) at elevated temperature.

## Results

### Metastable melting upon rapid heating

**Figure 1:**
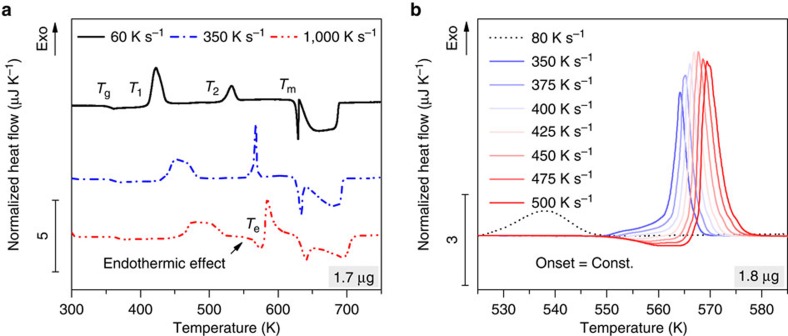
Heating of Au_70_Cu_5.5_Ag_7.5_Si_17_. (**a**) FDSC heat flow curves normalized to the heating rate. At low rates, a glass transition (*T*_g_), exothermic phase transition peaks (*T*_1_ and *T*_2_) and endothermic melting (*T*_m_) can be seen. The phase transition temperatures *T*_1_ and *T*_2_ shift to higher temperatures with increasing heating rate, and at sufficiently high rates an unexpected endothermic peak (at *T*_e_) arises after *T*_1_ and just before *T*_2_. (**b**) Close-up of the start of the endothermic effect and the second exothermic peak (*T*_2_). The onset of the endothermic effect shows no rate dependence, which indicates melting, whereas the *T*_2_ peak shifts to higher temperatures with increasing rates and changes its shape and size if located above *T*_e._

Figure 1a illustrates FDSC heating curves of small-scale Au_70_Cu_5.5_Ag_7.5_Si_17_ specimens, which have been amorphized *in-situ* via rapid cooling from 748 K at a rate of 5,000 K s^−1^. The FDSC curve at a heating rate of 60 K s^−1^ shows a glass transition (*T*_g_), two exothermic events (*T*_1_ and *T*_2_) and melting (*T*_m_) towards an equilibrium liquid. This behaviour agrees well with results for Au_70_Cu_5.5_Ag_7.5_Si_17_ BMGs measured via conventional DSC^16^, and the onset of melting corresponds to the deepest eutectic temperature reported for the Au–Si–Cu system^22^. Increasing the heating rate to 350 K s^−1^ shifts *T*_1_ and *T*_2_ to higher temperatures, which is commonly expected for the crystallization of supercooled liquids and most solid–solid transitions^23^. A further increase in the heating rate to 1,000 K s^−1^ clearly reveals the surprising occurrence of an endothermic effect at *T*_*e*_ (≈558 K) after the *T*_1_ peak and right before the *T*_2_ peak.

Figure 1b shows a close-up of the endothermic effect and the *T*_2_ peak at heating rates of 350–500 K s^−1^. While the peak at *T*_2_ shifts to higher temperatures when the rate increases, the endothermic effect shows no rate dependence, which is a common sign of melting. Note that *T*_e_ is far below *T*_m_, that is, the temperature *T*_e_ corresponds to that of a metastable liquid. This metastable liquid re-transforms very rapidly (within a few ms) towards a more stable crystalline state within the subsequent exothermic event at *T*_2_. For comparison, Fig. 1b also shows the *T*_2_ peak at a rate of 80 K s^−1^, where it clearly occurs below *T*_e_ (such that the metastable melting at *T*_e_ does not occur). Comparing the rates at 80 K s^−1^ and above 350 K s^−1^, it is obvious that the *T*_2_ peaks are remarkably different in shape and size.

If we examine the enthalpies of these transformations it becomes obvious that the crystallization enthalpy at *T*_1_ only exhibits a low heating rate dependency, whereas the enthalpy of the *T*_2_ peak is roughly 1/3 of the *T*_1_ peak if *T*_2_<*T*_e_ (≈558 K) but strongly increases to ≈3/2 of the *T*_1_ peak if *T*_2_>*T*_e_. (Figure 3b illustrates how the *T*_e_ endothermic peak is separated from the *T*_2_ exothermic peak.) Because the enthalpy of melting at *T*_m_ remains similar for all heating curves in Fig. 1, this simple energetic consideration indicates clearly that the Au-based BMG must transform through a metastable liquid upon heating, that is, glass → undercooled liquid → metastable crystalline state → metastable liquid → more stable crystalline state → equilibrium liquid. The metastable state appears to consist of one major metastable phase, which transforms via metastable melting (see also Supplementary Fig. 1) into stable primary crystals within an eutectic microstructure. The latter can be concluded from the melting event at *T*_m_, which contains one sharp endothermic peak (corresponding to eutectic melting), followed by a broader endothermic event (see Fig. 1a).

### Freezing the non-equilibrium states

**Figure 2:**
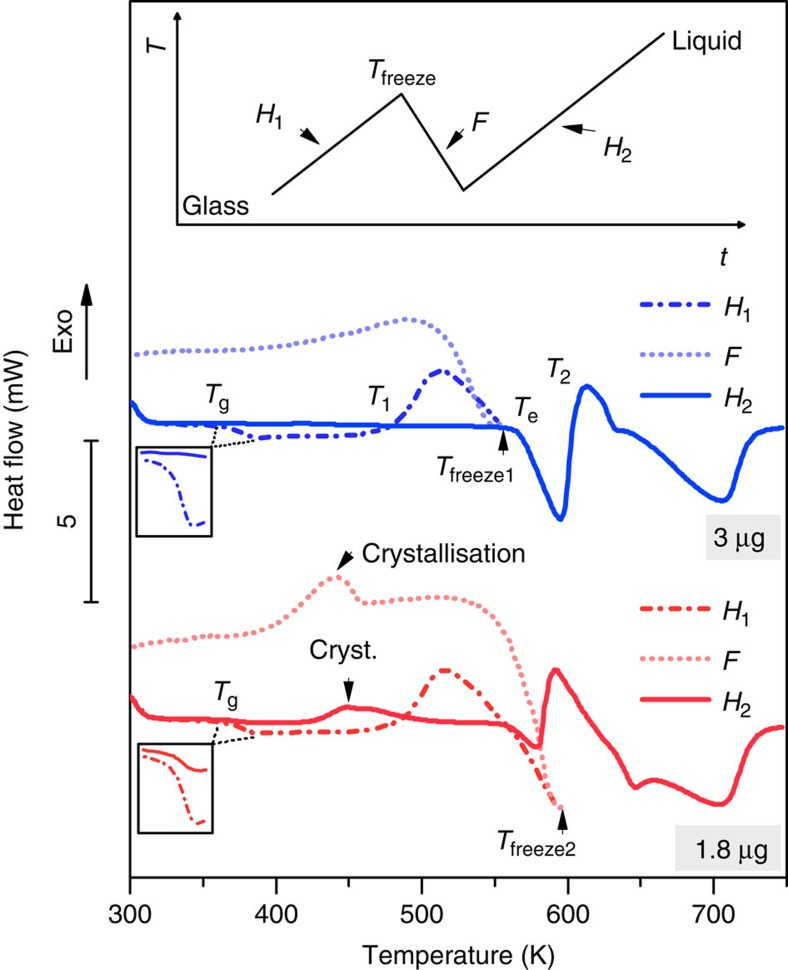
Freezing non-equilibrium states. Heat flow curves are shown after heating the Au_70_Cu_5.5_Ag_7.5_Si_17_ glass with a rate of 2,000 K s^−1^ (*H*_1_) to *T*_freeze(1,2)_ and cooling with a rate of 5,000 K s^−1^ (*F*) and subsequent heating with a rate of 2,000 K s^−1^ (*H*_2_); the upper image illustrates the applied time–temperature regime. The freezing temperature *T*_freeze1_ (blue curves) was chosen to be just above the formation of the metastable crystalline state (*T*_1_), while *T*_freeze2_ (red curves) was chosen to be within the metastable liquid. No reactions can be seen upon freezing the metastable crystalline state and no glass transition or crystallization is observed after re-heating with a rate of *H*_2_=2,000 K s^−1^ to *T*_freeze1_. This indicates that the material was mostly crystallized during the first heating step. Upon freezing from *T*_freeze2_ partial crystallization is observed, and upon re-heating with *H*_2_ a small glass transition and crystallization event are seen below *T*_freeze2_. This provides evidence that a metastable liquid has formed during the endothermic event at *T*_e_.

In the following we present further evidence for the unusual solid–solid phase transition via melting. We first prepared glassy Au_70_Cu_5.5_Ag_7.5_Si_17_ *in-situ* in the FDSC via rapid cooling from 748 K at a rate of 5,000 K s^−1^. We then processed the alloys according to the upper image in Fig. 2: after heating with *H*_1_=2,000 K s^−1^ to *T*_freeze(1,2)_, the specimens were cooled at a freezing rate *F* of 5,000 K s^−1^ to room temperature and then re-heated at *H*_2_=2,000 K s^−1^ to 748 K. The lower images in Fig. 2 show the corresponding heat flow curves. The blue dash-dotted curve indicates heating with *H*_1_ to *T*_freeze1_, which is above *T*_1_ but slightly below *T*_e_. In this case a glass transition occurs, followed by the formation of a metastable crystalline state at *T*_1_. Upon freezing with *F*=5,000 K s^−1^ (dotted blue curve) no further crystallization is observed and no glass transition detected. This implies that the supercooled liquid was mostly crystallized at *T*_freeze1_. Only upon reheating with *H*_2_=2,000 K s^−1^ (solid blue curve) are additional peaks observed above *T*_freeze1_, illustrating the melting of the metastable crystalline state at *T*_e_ and its subsequent re-crystallization at *T*_2_. If the material is frozen during the formation of the metastable liquid at *T*_freeze2_, partial crystallization occurs upon freezing (dotted red curve) and a small glass transition and a small first crystallization event are observed upon reheating (solid red curve). This is further proof that metastable melting is indeed observed at *T*_e_. However, the overlapping transition to a ‘stable' crystalline state at *T*_2_ prevents freezing to a fully amorphous state.

### Rate dependence of the phase transitions

**Figure 3:**
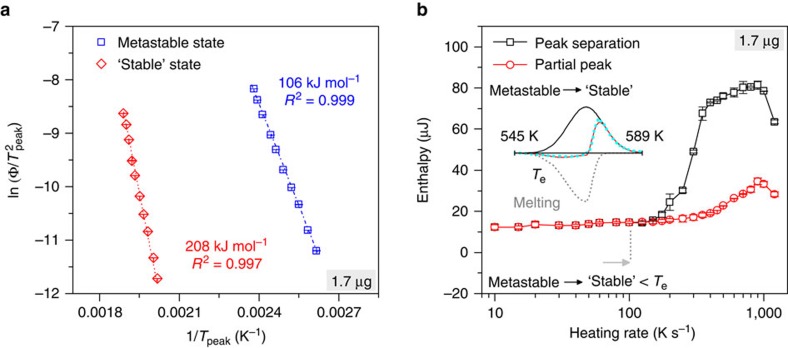
Rate dependence of phase transitions. (**a**) Arrhenius representation of the temperature dependence of Φ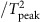, where Φ is the heating rate and *T*_peak_ is the corresponding peak temperature of crystallization (Kissinger plot). The crystallization towards the ‘stable' state reveals a large effective activation energy, indicating its kinetically difficult transition. *R*^2^ denotes the high quality of the fit. (**b**) Rate dependence of the transition enthalpy from the metastable to the ‘stable' state. The red curve shows the crystallization enthalpy 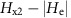 of the observed partial peak, using a horizontal baseline, and the black curve shows the mathematically separated individual crystallization enthalpy *H*_x2_. The transition enthalpies increase at a rate of 100 K s^−1^, where the transition overlaps with metastable melting. Above 1,000 K s^−1^ the enthalpies decrease again, because the formation of the ‘stable' state is then not yet completed at *T*_m_. The insert illustrates the applied peak separation at a heating rate of 600 K s^−1^, where the turquoise dots represent the FDSC data and the solid red line is the difference curve of the fitted metastable → ‘stable' transition (solid black line) and melting (grey dots). Error bars are defined as standard deviations.

We now turn to the rate dependence of the formation of the solids. Figure 3a shows a Kissinger plot^24^, from which the effective activation energies of the transitions can be deduced^17^. Such activation energies are a common measure of phase transformation kinetics^25^. As seen in Fig. 3a, the activation energy required to form the metastable crystalline state is lower by nearly a factor of two compared with that needed to form the ‘stable' state, which is thus much more difficult to form kinetically. This also supports the idea that it is easier for the metastable crystalline state to transform into a metastable liquid than into a ‘stable' crystalline state.

Figure 3b details the rate dependence of the transition enthalpy from the metastable to the ‘stable' crystalline state. Figure 1 revealed a Gaussian shape of the corresponding transition peak (*T*_2_) for temperatures <*T*_e_ (heating rate <100 K s^−1^), but a strong influence of the overlapping melting of the metastable state for temperatures >*T*_e_ (rate>100 K s^−1^), which also made the *T*_2_ peak much sharper for *T*>*T*_e_. To separate the melting peak from the *T*_2_ crystallization peak, the transition from metastable to ‘stable' was thus fitted with a Gaussian and, for melting of the metastable state, with a BiGaussian function. This is illustrated in the insert to Fig. 3b for a heating rate of 600 K s^−1^. The transition enthalpy increases above a rate of 100 K s^−1^, where the transition from metastable to ‘stable' starts to overlap with the formation of the metastable liquid at *T*_e_. This behaviour is so pronounced that it is still obvious if the transition peak resulting from the overlap of metastable melting and crystallization is integrated using a horizontal baseline. Note also that at high rates above 1,000 K s^−1^ the formation of the ‘stable' state cannot be completed kinetically before the temperature approaches *T*_m_, which is why the enthalpy decreases again. At even higher rates the melting peak at *T*_e_ no longer overlaps significantly with the *T*_2_ crystallization peak, which is illustrated in the Supplementary Fig. 1a for a heating rate of 3,000 K s^−1^. Here the melting of the metastable crystalline state becomes very obvious and can be clearly distinguished from a *c*_*p*_ step, which would result from an additional glass transition.

## Discussion

**Figure 4:**
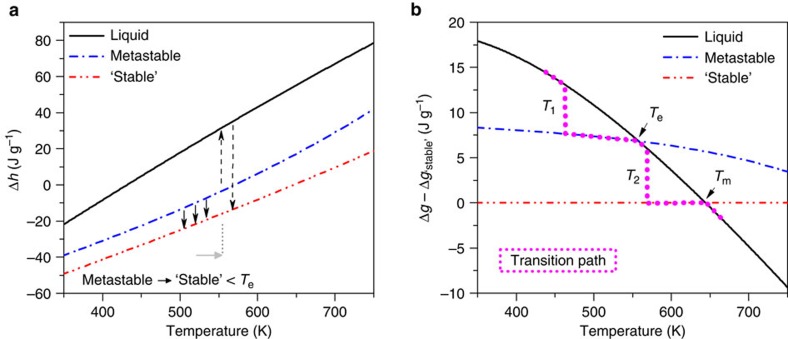
Thermodynamics of Au_70_Cu_5.5_Ag_7.5_Si_17_. (**a**) Specific enthalpies of the liquid, metastable crystalline state and ‘stable' crystalline state. The enthalpy changes due to transitions from the metastable to the ‘stable' state, including the formation of a metastable liquid, are shown by arrows. (**b**) Gibbs free energy diagram. The magenta dotted line reveals the transition path supercooled liquid → metastable crystalline state → metastable liquid → ‘stable' crystalline state → equilibrium liquid.

In the following, we discuss our FDSC data by evaluating the thermodynamics of the Au_70_Cu_5.5_Ag_7.5_Si_17_ BMG. The thermodynamic data are obtained from the specific heat capacities (*c*_*p*_) of the liquid and the metastable and ‘stable' states, and their corresponding transition enthalpies and temperatures. Conventional DSC was deployed to determine the transition temperatures and specific enthalpies, because of the relatively high uncertainty of the sample mass determination in FDSC. Specific heat capacities were measured by temperature-modulated DSC (TMDSC)^26^ and fitted by polynomials^27^,^28^ (provided in the Supplementary Discussion). The resulting DSC/TMDSC heat flow curves are comparable with the curves measured by FDSC at 60 K s^−1^, presented in Fig. 1a. The relative specific enthalpies, as shown in Fig. 4a, were calculated according to 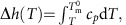 where 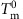 is the melting temperature of the ‘stable' state (more details are presented in the Supplementary Discussion). Figure 4a shows that at low heating rates only the small transition enthalpy from metastable to ‘stable' (small arrows pointing downwards) is observed, while at fast heating rates the transition enthalpies following the path metastable crystalline → metastable liquid → ‘stable' crystalline (dashed arrows) become visible. The values from Fig. 4a correspond well to the FDSC data in Fig. 3b, taking into account the uncertainty of the mass determination in FDSC (∼10%).

In this context it should be mentioned that we work with a four-component BMG to ensure sluggish transformation kinetics. The metastable crystalline state may thus contain more than one phase, but the transformation enthalpies measured reveal clearly that the majority of the material follows the proposed transition path. In fact, the FDSC data of the Supplementary Fig. 1 and the melting events shown in Fig. 1a indicate that this major metastable phase transforms via metastable melting to stable primary crystals within an eutectic microstructure. Future transmission electron microscopy studies may further illustrate these microstructural features.

Figure 4b shows the relative Gibbs free energies (Δ*g*) of the various states, which can be calculated from 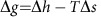, where 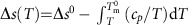 is the relative entropy and Δ*s*^0^ is approximated by Δ*h*^0^/*T*^0^, with Δ*s*^0^, Δ*h*^0^ and *T*^0^ as the entropy, enthalpy and temperature of the solid-to-equilibrium liquid transition. All data for the ‘stable' state and liquid are accessible via DSC and TMDSC, while the temperature of metastable melting (*T*_e_≈558 K) is estimated from FDSC and the corresponding specific enthalpy (35.1 J g^−1^) is read from Fig. 4a. For better visualization Fig. 4b plots the relative Gibbs free energy with reference to the ‘stable' state. The mangenta dotted line schematically illustrates the kinetic transition path of the solid–solid transition via melting. Starting from the supercooled liquid, Δ*g* is abruptly reduced at *T*_1_ by the formation of the metastable crystalline state. The kinetically difficult solid–solid transition to the ‘stable' state (see also Fig. 3a) enables the lowering of Δ*g* via the formation of a metastable liquid at *T*_e_. Figure 4b thus illustrates that there is a thermodynamic driving force^9^ for the formation of a metastable liquid, and from refs 8, 9 it can be concluded that the liquid–solid interfacial free energy is likely to be lower than that between the solids. This triggers the transition to the ‘stable' state at *T*_2_, where Δ*g* is again abruptly lowered. Finally, the transition to the equilibrium liquid occurs at *T*_m_.

We have thus documented the discovery of a solid–solid transition via melting in a metallic system. The Au_70_Cu_5.5_Ag_7.5_Si_17_ BMG exhibits the following unusual transition path upon heating: glass → undercooled liquid → metastable crystalline state → metastable liquid → ‘stable' crystalline state → equilibrium liquid. With FDSC we are able to explore this new transition path *in-situ* and, using also conventional DSC, gain new insights into the underlying thermodynamics.

In conclusion, we suggest that the observed fundamental solid–solid transition via metastable melting is a feature of many metallic glasses and metals, where different formation sequences of solid phases with varying thermodynamic stability are frequently observed^29^. The concept presented can be applied to any metastable to more stable transformation of crystals and does not require the glassy state. This is illustrated in Supplementary Fig. 1b, where a metastable crystalline state can transform into a ‘stable' crystalline state even if no glass has formed beforehand upon slow cooling. The possibility of studying solid–solid transitions via melting in real atomic systems demonstrated here is thus expected to provide further insight into phase transition theory, and may also trigger the development of new materials. For example, FDSC may be deployed to gain a deeper understanding of the reversion of precipitates upon heating in conventional metallurgy^30^. Finally, the concept presented may also be of great importance for the understanding of new processing techniques, involving metals and BMGs, where rapid cooling and heating are applied (for example, 3D printing of metals) and metastable phases form frequently^31^.

## Methods

### Sample preparation

The elements Au (purity 99.99 wt%), Ag (99.99 wt%), Si (99.999 wt%) and Cu (99.995 wt%) were weighed according to the atomic composition Au_70_Cu_5.5_Ag_7.5_Si_17_ and inserted into quartz glass tubes of 5 mm diameter. After repeated purging with Ar (5N purity), the tube ends were closed under 200 mbar Ar pressure and the element pieces were induction-melted to a pre-alloy. From this pre-alloy, thin (∼30 μm) and chemically homogeneous glassy ribbons were produced by melt spinning under a 500 mbar He atmosphere (5N purity).

### Fast differential scanning calorimetry

FDSC was performed in power compensation mode using a Mettler-Toledo Flash-DSC 1. The sample support temperature of the FDSC was set to 183 K using a Huber intracooler TC90. The FDSC samples were prepared by cutting the melt-spun ribbons into small pieces under a stereomicroscope and then transferred using an electrostatic manipulator hair onto a conditioned and temperature-corrected MultiSTAR UFS1 sensor. The sample masses were estimated to be 1.7 to 3 μg according to the procedure given in ref. 18. Reproducibility was checked by comparing the same thermal cycles at the start and end of each measurement series.

### Conventional and temperature-modulated DSC

Conventional DSC was performed using a differential scanning calorimeter (Mettler-Toledo DSC1) to determine the temperatures and enthalpies of the transitions. The measurements were conducted at a heating rate of 0.16 K s^−1^ using aluminium pans on the sample and reference platforms. Heat flow calibration was performed with indium and zinc.

Specific heat capacity measurements were performed using TMDSC in a Mettler-Toledo DSC1 equipped with TOPEM stochastic temperature modulation^26^. Sapphire was used as reference substance for the specific heat capacity determination. The measurements were conducted at heating and cooling rates of 0.033 K s^−1^ and a temperature modulation of 2 K, using aluminium pans on the sample and reference platforms. Data processing was performed with a calculation window width of 300 s and a corresponding shift of 10 s. For the specific heat capacity determination of the liquid phase, heating and cooling measurements were combined to increase the temperature range of the measured data.

## Additional information

**How to cite this article:** Pogatscher, S. *et al.* Solid–solid phase transitions via melting in metals. *Nat. Commun.* 7:11113 doi: 10.1038/ncomms11113 (2016).

## Supplementary Material

Supplementary Information Supplementary Figure 1 and Supplementary Discussion
