# Supplementary material for: Solid–solid phase transitions via melting in metals
Source: Nat Commun. 2016 Apr 22;7:11113. doi: 10.1038/ncomms11113 (PMC4844691; doi:10.1038/ncomms11113)
Supplement: Supplementary Information — Supplementary Figure 1 and Supplementary Discussion [file ncomms11113-s1.pdf]

Supplementary FDSC data

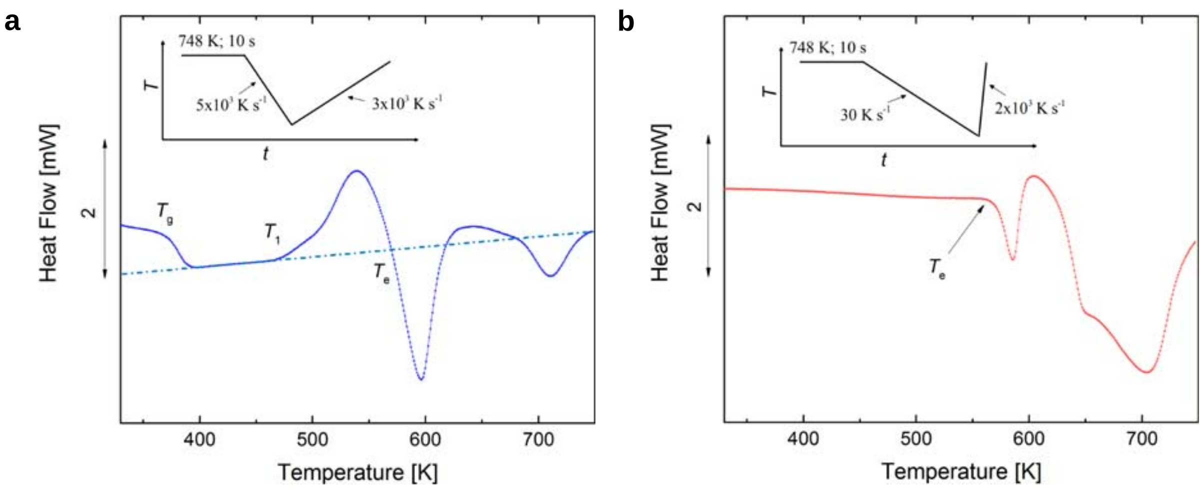

Supplementary Figure 1 | Supplementary FDSC data. a, FDSC heat flow curve of $Au_{70}Cu_{5.5}Ag_{7.5}Si_{17}$ normalized to the heating rate for a heating rate of 3000 K s^{-1} after in-situ amorphization via rapid cooling from 748 K at a rate of 5000 K s^{-1}. A significant amount of the metastable crystalline state is formed during heating, which transforms to the metastable liquid at T_e. Heating at a rate of 3000 K s^{-1} is fast enough to reach the temperature of the equilibrium melt before much of the “stable” crystalline state can form from the metastable liquid. Hence, the melting peak at T_e is no longer hidden by the formation of the more stable crystalline state, such that the formation of the metastable liquid is obvious and clearly distinguishable from a c_p-step resulting from an additional glass transition. The straight line serves as a guide to the eye. **b,** FDSC heat flow curve of $Au_{70}Cu_{5.5}Ag_{7.5}Si_{17}$ normalized to the heating rate for a heating rate of 2000 K s^{-1} after slow cooling from 748 K at a rate of 30 K s^{-1}. Because the rate of 30 K s^{-1} is far below the critical cooling rate of $Au_{70}Cu_{5.5}Ag_{7.5}Si_{17}$, a fully crystalline state is obtained, as indicated by the missing glass transition in the FDSC trace upon heating. The formation of a metastable liquid at T_e and the usual melting of the “stable” crystalline state illustrate that a metastable state can also transform to a more stable state via melting even if no glass has formed beforehand. For such high heating rates the thermal lag of the FDSC device can generate a smearing of the peaks, as seen for the melting of the “stable” state.

Supplementary Discussion

Thermodynamics of $Au_{70}Cu_{5.5}Ag_{7.5}Si_{17}$

Specific heat capacities were measured via TMDSC, and the specific heat capacities of the liquid, the metastable state and the "stable" state are described by the polynomials

$$c_{p,\text{liquid}} = 0.340\ \text{J g}^{-1}\text{K}^{-1} - 1.46\times10^{-4}\ \text{J g}^{-1}\text{K}^{-2}\ T - 2402\ \text{J g}^{-1}\text{K}\ T^{-2},$$

$$c_{p,\text{metastable}} = 0.154\ \text{J g}^{-1}\text{K}^{-1} - 1.24\times10^{-4}\ \text{J g}^{-1}\text{K}^{-2}\ T + 3.67\times10^{-7}\text{J g}^{-1}\text{K}^{-3}\ T^{2}, \text{ and} \quad (1)$$

$$c_{p,\text{"stable"}} = 0.151\ \text{J g}^{-1}\text{K}^{-1} - 7.69\times10^{-6}\ \text{J g}^{-1}\text{K}^{-2}\ T + 7.42\times10^{-8}\ \text{J g}^{-1}\text{K}^{-3}\ T^{2}.$$

For the "stable" state and the liquid the relative specific enthalpies can be directly calculated according to equations 2 and 3:

$$\Delta h_{\text{"stable"}}(T) = h_{\text{"stable"}}(T) - h_{\text{"stable"}}(T_\text{m}^0) = -\int_T^{T_\text{m}^0} c_{p,\text{"stable"}}\text{d}T\ , \quad (2)$$

$$\Delta h_{\text{liquid}}(T) = h_{\text{liquid}}(T) - h_{\text{"stable"}}(T_\text{m}^0) = \Delta h_\text{m}^0 - \int_T^{T_\text{m}^0} c_{p,\text{l}}\text{d}T, \quad (3)$$

where T_m^0 is the melting temperature of the "stable" state (645 K) and Δh_m^0 is the specific enthalpy of melting (54.3 J g^{-1}). With the formation enthalpy of the metastable state at 375 K (19.9 J g^{-1}), accessible from DSC measurements, the relative specific enthalpy of the metastable state $(\Delta h_\text{metastable})$ can be calculated from these data according to equation 4:

$$\Delta h_{\text{metastable}}(T) = h_{\text{metastable}}(T) - h_{\text{"stable"}}(T_\text{m}^0) = 19.9\ \text{J g}^{-1} - \int_T^{375\ \text{K}} c_{p,\text{metastable}}\ \text{d}T + \Delta h_{\text{"stable"}}(375\ \text{K}). \quad (4)$$

Note that the transition enthalpy from the metastable to the "stable" state read from the enthalpy curves in Fig. 4a at 478 K agrees well with values experimentally determined via conventional DSC (9.8 J g^{-1}).

The relative specific Gibbs free energies of the "stable" state and the liquid are calculated using equations 5 and 6, respectively:

$$\Delta g_{\text{"stable"}}(T) = g_{\text{"stable"}}(T) - g_{\text{"stable"}}(T_{\mathrm{m}}^{0}) = \Delta h_{\text{"stable"}}(T) + T \int_{T}^{T_{\mathrm{m}}^{0}} \frac{c_{p,\text{"stable"}}}{T} \mathrm{d}T \,, \tag{5}$$

$$\Delta g_{\text{liquid}}(T) = g_{\text{liquid}}(T) - g_{\text{liquid}}(T_{\mathrm{m}}^{0}) = \Delta h_{\text{liquid}}(T) - T\left(\Delta s_{\mathrm{m}}^{0} - \int_{T}^{T_{\mathrm{m}}^{0}} \frac{c_{p,\text{liquid}}}{T} \mathrm{d}T \right). \tag{6}$$

Calculating the relative specific Gibbs free energy of the metastable state is impossible using only DSC and TMDSC data, because of the unknown melting temperature of the metastable state. Using FDSC we estimate this temperature to be $T_{\mathrm{e}} \approx 558$ K, and the enthalpy of melting is read from Fig. 4a to be $\Delta h_{\text{metastable}}^{0} \approx 35.1$ J g^{-1}. The relative specific Gibbs free energy of the metastable state can then be calculated according to equation 7:

$$\Delta g_{\text{metastable}}(T) = g_{\text{metastable}}(T) - g_{\text{"stable"}}(T_{\mathrm{m}}^{0}) = \Delta h_{\text{metastable}}(T) - T\left(\Delta s_{\text{"stable"}}(T_{\mathrm{e}}) + \frac{\Delta h_{\text{metastable}}^{0}}{T_{\mathrm{e}}} - \int_{T}^{T_{\mathrm{e}}} \frac{c_{p,\text{liquid}}}{T} \mathrm{d}T \right).$$

(7)
